# Danazol: An Effective and Underutilised Treatment Option in Diamond-Blackfan Anaemia

**DOI:** 10.1155/2019/4684156

**Published:** 2019-07-01

**Authors:** Kern Y. Chai, Cara J. Quijano, Shingirai Chiruka

**Affiliations:** Department of Haematology, Dunedin Hospital, 201 Great King Street, Dunedin 9016, New Zealand

## Abstract

Diamond-Blackfan anaemia (DBA) is a rare congenital red cell aplasia that presents in infancy. The exact molecular mechanism of ineffective erythropoiesis and red cell aplasia remains unclear, rendering targeted therapy elusive. The mainstay treatment of DBA is with regular blood transfusion and long-term corticosteroids, both of which have long-term side effects. We report a case of DBA successfully treated with danazol, a synthetic androgen, and suggest that danazol be considered as a viable option in patients who become refractory to steroids and are considered high risk or unfit for allogeneic stem cell transplantation.

## 1. Introduction

Diamond-Blackfan anaemia (DBA) is a rare congenital bone marrow failure syndrome characterised by an isolated macrocytic anaemia that presents in infancy. The mainstay treatment of DBA is with regular blood transfusion with long-term corticosteroids, both of which have long-term side effects. Steroid refractory or intolerant patients may benefit from an allogeneic stem cell transplant, but it is associated with high mortality and morbidity. Danazol has been shown in previous case reports to be a safe treatment option with reasonable efficacy in DBA. We suggest that danazol be considered as an option for patients refractory to steroids or as a steroid-sparing agent in DBA.

## 2. Case Report

A 33-year-old New Zealand European male was suspected of having DBA when he was two months old. He was taken to his general practitioner for a routine 6-week check, during which he was noted to be pale, and a subsequent full blood count revealed an isolated macrocytic anaemia (Hb 94 g/L). Of relevance, his mother was diagnosed with pure red cell aplasia when she was 14 months old, raising the possibility of a congenital bone marrow failure syndrome. A bone marrow biopsy revealed markedly reduced erythroid precursors consistent with DBA. He was placed on prednisone from two months of age. In addition to treatment with a corticosteroid, he required multiple blood transfusions between the ages of two months and two years. At four and a half years of age, oxymetholone was trailed as adjunctive therapy to prednisone at a total daily dose of 25 mg (2 mg/kg) to allow a steroid taper but was ineffective. A trial of danazol or cyclosporine was considered but was never undertaken due to lack of evidence regarding its efficacy in DBA. The patient's erythropoietin (EPO) levels were evaluated at the age of six and found to be raised at 197 *μ*M/ml (normal range 5–53 *μ*M/ml).

Later in life, it was noted that the patient had several physical malformations associated with DBA. He had dysmorphic features of short stature, micrognathia, small hands with long fingers, and a slightly widened neck. An echocardiogram revealed no cardiac abnormalities. When he was 12 years of age, he required orchidopexy for an undescended testis. Latterly, he was assessed by the endocrine service who concluded he was eugonadal, and his short stature was related to his long-term steroid use. As a result, attempts were made to reduce the prednisone dose slowly to the most tolerated but effective dose. He was stable for a number of years on between 7.5 mg daily and 7.5 mg on alternate days. He was transfusion independent between the ages of 2 and 32.

In November 2017, he presented with a profound hypoproliferative anaemia (Hb 47 g/L, reticulocyte 2 × 10^9^). He denied any new medications or bleeding. At the time, his prednisone dose was 7.5 mg/5 mg on alternate days. Investigations for alternate causes of anaemia were unrevealing, with pertinent negatives including an unremarkable blood film, normal haematinics (B12 263 pmol/L, folate 36.4 nmol/L, and iron 23 *μ*mol/L), negative haemolysis screen, and negative parvovirus, EBV, and CMV serology and PCR. Ferritin and transferrin saturation at the time were slightly elevated (737 *μ*g/L and 92%, respectively), and transferrin was slightly decreased (1 g/L) likely secondary to ineffective erythropoiesis. A repeat bone marrow showed a hypocellular trephine with relatively preserved macronormoblastic erythropoiesis. There was no evidence of dysplasia, disease progression, or transformation. Cytogenetics revealed a normal karyotype. Finally, genetic testing was sent to Oxford University Hospital in the United Kingdom which revealed a point mutation in intron 2 of *RPS19*, confirming the diagnosis of DBA.

One month later, his prednisone was increased up to 2 mg per kg (totalling 110 mg per day) after requiring 7 units of RBCs within the preceding month. He remained on this dose of prednisone for three months, achieving a peak haemoglobin level of 84 g/L, but relapsed into transfusion dependency each time his prednisone was tapered. By this stage, the patient had cumulated many side effects related to his medical therapy. The long-term steroid use led to gastritis, osteoporosis, hypokalaemia, and central serous retinopathy requiring further co-treatment with omeprazole, alendronic acid, and cholecalciferol. He also developed transfusional haemosiderosis with a peak ferritin level of 1946 *μ*g/L and transferrin saturation of 96%, necessitating chelation therapy with deferiprone. This was the first time that the patient had required a chelating drug. Unfortunately, he developed profound neutropenia from the deferiprone which culminated in a prolonged admission with extensive lower limb cellulitis, and the deferiprone was permanently discontinued. A cardiac MRI did not show significant iron overloading.

**Figure 1:**
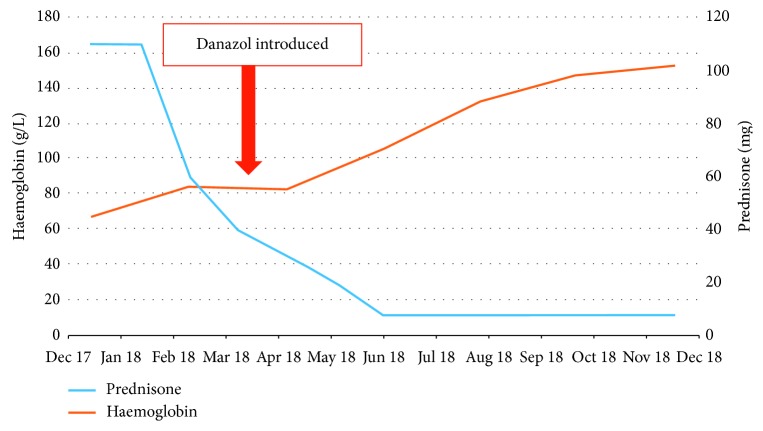
Line graph depicting improvement in haemoglobin despite decreasing prednisone dose following the introduction of danazol.

Given the toxicities, alternate treatment options were re-discussed including danazol, metoclopramide, and allogeneic stem cell transplant (AlloSCT). He was worked up for a transplant but did not have any matched sibling donors, and he was also considered as high risk for allogeneic stem cell transplantation given his age and multiple prior RBC transfusions. Thus, the decision was made to commence danazol 200 mg daily in March 2018. A starting dose of 200 mg was chosen as it is a standard starting dose for most indications. At this time, he was taking 60 mg of prednisone a day. Following the introduction of danazol, his haemoglobin rose, allowing the prednisone dose to be decreased (Figure 1, Table 1). Over the following few months, he had been weaned down to 7.5 mg of prednisone daily which he is taking in addition to the 200 mg of danazol while maintaining a haemoglobin level of 148–160 g/L six months later. The patient has been left on the minimum effective dose of danazol. His liver function tests have remained normal while on danazol.

## 3. Discussion

### 3.1. Introduction, Epidemiology, and Clinical Presentation

DBA is a rare congenital ribosomopathy characterised by erythroid hypoplasia, developmental malformations, and an increased risk of malignancy. The vast majority of patients are diagnosed within the first year of life with an incidence rate of 5–7 : 1,000,000 live births [1, 2]. Patients typically present with severe isolated macrocytic anaemia with reticulocytopenia. Bone marrow findings are usually normocellular with absent erythroid precursors [3]. Approximately half of all patients will have maxillofacial, thumb, cardiac, or renal malformations [2, 4]. There is an approximate 20% cumulative incidence of malignancies by the age of 50 [5, 6].

### 3.2. Pathogenesis and Inheritance

DBA is inherited in autosomal dominant fashion but with incomplete penetrance. Over 50% of DBA cases have somatic mutations or deletions in one of 14 genes encoding for ribosomal proteins, with the *RPS19* gene being the commonest genetic abnormality accounting for a quarter of cases [4, 7–9]. Genotype-phenotype correlations have been observed with *RPL5* and *RPL11* mutations associated with higher rates of physical malformations [8]. The exact molecular mechanism by which ribosomal dysfunction leads to ineffective erythropoiesis and red cell aplasia remains unclear. Dutt et al. found that haploinsufficiency of ribosomal protein genes activates the p53 pathway, culminating in preferential accumulation of p21 in erythroid progenitor cells and consequent cell cycle arrest [10]. Other postulated mechanisms relate to defective erythroblast mRNA generation or altered haeme metabolism [9, 11, 12]. Further empiric proof is evidenced by rescued erythropoiesis with pharmacological inhibition of p53 in both human and mouse models [13, 14].

In relation to our case, the patient was found to be heterozygous for a G to A nucleotide substitution at the first nucleotide in intron 2 of *RPS19* (*RPS19* C. 71 + 1G > A). The testing was undertaken at Oxford University Hospital using a combination of Sanger sequencing and next generation sequencing involving custom design amplicons run on the Illumina MiSeq. This mutation has been previously reported to be associated with DBA [15].

The patient's mother was diagnosed with red cell aplasia when she was 14 months of age. She was treated initially with cortisol and then switched to prednisone at the age of two in addition to multiple blood transfusions. She went into remission between the ages of two and three. The anaemia reoccurred when she was six years old, and she was placed back on prednisone until she once again went into remission at the age of 12. Since her second remission, she has not required further treatment. The other members of the patient's family, including maternal grandparents, have not shown symptoms of DBA. Therefore, this may be a de novo mutation that has arisen in the mother. The mother has not been tested for the mutation found in the patient.

### 3.3. Treatment

The mainstay treatment of DBA is with regular RBC transfusion and long-term corticosteroids [2, 3]. RBC transfusion intervals are typically every 4–6 weeks, and the cumulative iron loading can lead to significant endocrine, cardiac, hepatic, and joint dysfunction. The transfusional hemosiderosis can be minimised by chelation therapy but is usually poorly tolerated. Maintenance corticosteroids, whilst effective in about 80% of patients, have led to significant long-term toxicities causing a cessation in therapy in about half of patients [16].

Steroid refractory or intolerant patients may benefit from an AlloSCT [2, 7, 16]. Long-term registry data indicate 40% of patients are steroid dependent, 40% are transfusion dependent due to poor response or intolerance to steroids, and the remaining 20% are in haematologic remission (either spontaneous or post-AlloSCT) [17, 18]. Other treatments have been trialled in a small number of patients with minimal success. Immunosuppressive therapies including IVIG and cyclosporine have been used despite limited theoretical basis [1, 3, 7, 16]. Metoclopramide has been trialled on the basis of increasing prolactin levels to stimulate the erythroid precursor microenvironment, but with disappointing results [19]. Targeted therapy in the form of leucine remains experimental [2]. Clearly, there is a need for more effective and safer treatments that provide long-term control.

### 3.4. Danazol

Danazol is a synthetic androgen compound that inhibits pituitary gonadotrophins. It also has mild immunosuppressive properties via inhibition of TNF-alpha and IL-1 [20]. Its side effect profile is unsurprisingly related to hyperandrogenism but is also rarely associated with the development of hepatocellular carcinoma (HCC) and hepatocellular adenomas [21, 22].

Polycythaemia is a common side effect observed in testosterone-treated men, whereas haemoglobin levels in women with congenital adrenal hyperplasia also correlate with androgen levels [23]. More recently, testosterone has been shown to increase erythropoietin (EPO) levels while concurrently reducing hepcidin levels, therefore suggesting a molecular basis for androgen-induced erythropoiesis [24]. In addition, it has also been utilised to overcome EPO-resistance in renal-mediated anaemia [25, 26]. It stands to reason that danazol, a synthetic androgen, could also produce such effects in vivo leading to a targeted proliferation of erythroid precursors via the JAK/STAT signalling pathway [27].

Danazol has also shown activity in treating anaemia in other haematological conditions including aplastic anaemia, myelodysplasia, myelofibrosis, pure red cell aplasia, and other inherited bone marrow failure syndromes including Fanconi anaemia and dyskeratosis congenita [19, 28–33]. Jaime-Pérez et al. studied a cohort of 50 patients with aplastic anaemia of which 37 were treated with danazol. They found a response rate of 46% with a 41% five-year overall survival in the patients treated with danazol (compared with 92% in the remaining patients receiving allogeneic haematopoietic stem cell transplantation) [20]. Scheckenbach et al. collected data on eight patients with Fanconi anaemia who had been treated with danazol. They found that seven of the patients responded with marked improvement in both haemoglobin and platelets [31].

The patient had a haematological response to danazol, but a trial of oxymetholone earlier in life was found to be ineffective despite the fact that these medications are both androgens. We hypothesize that this may be due to the agonistic effects of danazol on glucocorticoid receptors, resulting in danazol having bimodal upregulating effects on erythropoiesis [34].

### 3.5. Relevance to Case

The patient developed steroid-resistant DBA after being on low-dose prednisone for 30 years, resulting in transfusion dependency. The addition of danazol led to a complete haematologic response sustained at 12 months (at time of writing) that was not seen even with salvage high-dose prednisone (2 mg/kg). The patient has significant toxicity from the chronic corticosteroid treatment including osteoporosis and central serous retinopathy in his 30s. In addition, he had life-threatening neutropenic sepsis from deferiprone, which was introduced when he became transfusion dependent after the prednisone response was lost. His prednisone is being tapered with the aim of transitioning to danazol monotherapy in the future. Whilst danazol is not completely benign (increased risk of HCC), it clearly has less risk and morbidity compared to long-term corticosteroids or an AlloSCT.

Other case reports highlighting the efficacy of danazol have been published. Flores Ballester et al. described a patient with a congenital hypoplastic left thumb who developed a hypoproliferative anaemia in his 30s [35]. He was diagnosed with adult-onset DBA after the identification of a pathogenic mutation in *RPL11*. He was unresponsive to steroids and became transfusion dependent. He was commenced on cyclosporine with minimal improvement until the addition of danazol (400 mg per day). The patient remains in haematologic remission for the last 16 months since starting danazol monotherapy.

## 4. Conclusions

Danazol is a relatively underutilised treatment in DBA with a plausible targeted molecular basis for overcoming erythroid hypoplasia. It is cheap and relatively nontoxic compared to long-term corticosteroids, immunosuppressive therapy, or AlloSCT. Whilst large prospective randomised controlled trials to confirm the efficacy of Danazol in DBA is not realistic due to the rarity of this condition, directed laboratory studies could be carried out as a surrogate to ascertain its utility in DBA patients.

## Figures and Tables

**Table 1:** Depicting figures of haemoglobin and prednisone as shown in Figure 1. Haemoglobin figures stated in November 2017 are pre- and posttransfusion.

Date	Nov 17	Nov 17	Dec 18	Feb 18	Apr 18	Jun 18	Aug 18	Oct 18	Dec 18
Haemoglobin (g/L)	47	77	67	84	83	105	133	148	153
Prednisone (mg)	7.5/5	110	110	60	30	7.5	7.5	7.5	7.5
